# Case–Control Study of Risk Factors for Hospitalization Caused by Pandemic (H1N1) 2009 

**DOI:** 10.3201/eid1708.100842

**Published:** 2011-08

**Authors:** Kate A. Ward, Paula J. Spokes, Jeremy M. McAnulty

**Affiliations:** Author affiliation: New South Wales Department of Health, Sydney, New South Wales, Australia

**Keywords:** viruses, influenza A virus, H1N1, pandemic (H1N1) 2009, risk factors, hospitalization, outbreaks, case–control study, research, Australia

## Abstract

We conducted a case–control study to identify risk factors for hospitalization from pandemic (H1N1) 2009 virus infection among persons >16 years of age in Sydney, Australia. The study comprised 302 case-patients and 603 controls. In a logistic regression model, after adjusting for age and sex, risk factors for hospitalization were pregnancy (odds ratio [OR] 22.4, 95% confidence interval [CI] 9.2–54.5), immune suppression (OR 5.5, 95% CI 2.8–10.9), pre-existing lung disease (OR 6.6, 95% CI 3.8–11.6), asthma requiring regular preventive medication (OR 4.3, 95% CI 2.7–6.8), heart disease (OR 2.3, 95% CI 1.2–4.1), diabetes (OR 3.8, 95% CI 2.2–6.5), and current smoker (OR 2.0, 95% CI 1.3–3.2) or previously smoked (OR 2.0, 95% CI 1.3–3.0). Although obesity was not independently associated with hospitalization, it was associated with an increased risk of requiring mechanical ventilation. Public health messages should give greater emphasis on the risk for severe disease among pregnant women and smokers.

## Abstract 2

TOC summary: Pregnancy, asthma, diabetes, and a history of smoking were among risk factors found.

The emergence of pandemic (H1N1) 2009 virus (*1*,*2*) was associated with a large increase in the number of persons requiring hospitalization for severe influenza disease in many parts of the world (*3*–*5*). In response, in an effort to reduce the impact of the pandemic on their communities and health services, public health agencies developed recommendations for persons at increased risk for disease to seek early treatment. However, these recommendations were based on studies of seasonal influenza (*6*–*10*) and descriptive case reports (*11*–*20*).

The first cases of pandemic (H1N1) 2009 infection from New South Wales (NSW) were reported in May 2009. In NSW, laboratories were required to notify all pandemic (H1N1) 2009 diagnoses to the NSW Department of Health under Public Health Act 1991 (*21*). Australian public health management protocols recommended laboratory testing for all persons with influenza-like illness (fever and cough or sore throat) admitted to a hospital (*22*). Public health follow-up was required to ascertain hospitalization status for all notified cases at the time of diagnosis; this information was collated on a statewide database. Within Sydney, the capital city of NSW (population 4.4 million), there are 4 Area Health Services (AHSs) responsible for the provision of local public health and clinical services.

By the end of June 2009 (before a vaccine was available), community transmission was widespread in Australia, and public health efforts were focused on protecting those at greatest risk for severe disease. The groups considered most vulnerable were pregnant women; indigenous people; very obese persons; persons with pre-existing chronic medical conditions, including lung, heart, and kidney disease; and persons with blood, metabolic or neurologic disorders, immunosuppressive conditions, or asthma (*23*). Persons in these risk groups and those with severe disease were urged to seek medical attention early if influenza like symptoms appeared. Doctors were provided with free antiviral medication for patients who were seen within 48 hours of symptom onset. A vaccine became publicly available for distribution in September 2009. To help reach populations who would most benefit from prevention and early intervention, we sought to identify independent risk factors for moderate to severe disease from pandemic (H1N1) 2009 infection among adults and to describe the characteristics of those who sought early medical treatment to determine the effectiveness of public health messages.

## Methods

The study population was defined as persons >16 years of age residing in metropolitan Sydney during July 1–August 31, 2009. All interviews were conducted during September and October 2009.

### Cases

We defined a case as a person with influenza-like illness admitted (for a minimum of an overnight stay) to a Sydney metropolitan hospital from July 1 through August 31, 2009, who had laboratory confirmation of pandemic (H1N1) 2009 by PCR that was notified to the Department of Health. Patients <16 years of age or residing outside metropolitan Sydney were excluded from the study because we assumed the threshold for hospital admission may have been different for this age group and for patients in regional and rural areas. To ensure complete case ascertainment, all cases notified in the study period were cross-matched with the Department of Health database containing all hospital admissions in NSW for the study period; additional identified cases were included in the study. If case-patients were not able to complete a telephone interview because of ill health or disability, another household member completed the interview on their behalf. Up to 30 attempts were made to contact case-patients by telephone.

### Case–Control Study

In a case–control study, we compared demographic and health information reported by case-patients with those of controls. We defined a control as a person >16 years of age residing in metropolitan Sydney who had not been hospitalized for influenza in 2009. Telephone numbers (stratified by AHS) were used to randomly select potential control households. Within each AHS in Sydney, we selected 2 households per case. A single control was randomly selected from within each selected household for interview. Up to 12 attempts were made to contact selected households. Fewer attempts were made to contact controls than case-patients as a result of random-digit generation methods described in detail elsewhere (*24*). The age and sex distribution of participating controls was compared with that of the metropolitan Sydney population.

### Study Questionnaire

We used a standard questionnaire to ask case-patients and controls about influenza symptoms, pregnancy or delivery within the previous 28 days, weight and height, smoking history, current and previous medications, and past hospitalizations. In addition, information was collected regarding diagnosis of general health conditions, including asthma, lung disease (defined as emphysema, chronic lung problems and/or chronic bronchitis), heart disease (defined as heart problems from birth, rheumatic heart disease, angina, heart attack, and/or heart failure excluding hypertension), diabetes (type I or II), other metabolic disorders, kidney disease (defined as kidney transplant, renal failure, and/or dialysis), liver disease, blood disorders (defined as sickle cell disease, thalassemia, or hemoglobin problems), mental health diagnoses, neurologic conditions (defined as conditions that involve muscles, nerves, or the brain), immune suppression (defined as cancer, HIV infection, or immunosuppressive medication), and obstructive sleep apnea. Ethics approval was not required because information was collected under NSW Public Health Act 1991 (*21*)

### Statistical Analyses

In univariate analysis, the proportions of characteristics among case-patients and controls were compared by using χ^2^ tests. In multivariate analysis, independent risk factors for hospitalization were assessed through logistic regression by using backward elimination. All variables with a univariate level of significance p<0.25 were selected for inclusion in the base model, and variables were excluded if the p value was >0.05 and did not meaningfully alter the point estimates of the remaining variables. Because a similar proportion of case-patients and controls reported a diagnosis of asthma not requiring regular medication, only a history of asthma requiring regular medication was included in the final logistic regression model.

Additional logistic regression models were constructed to compare case-patients with controls for women of child-bearing age (16–45 years), case-patients who received mechanical ventilation compared with all controls, case-patients who sought medical attention within 48 hours of onset of symptoms compared with case-patients who sought medical attention after 48 hours, and controls who reported symptoms of influenza-like illness during the study period (defined as self-reported fever and either cough or sore throat) with controls who reported no illness during the study period. Mechanical ventilation (rather than intensive care unit admission) was used as a measure of severity of illness because cases were reported from several hospital facilities with varying criteria for patient admission to intensive care. The model fit the data well by the Hosmer-Lemeshow goodness-of-fit test (χ^2^ = 15.85, 9 df, p = 0.07). Seventy-nine percent of pairs were concordant, and *c* = 0.804. 

## Results

In total, 402 hospitalized patients were identified as eligible for inclusion in the study. Of these, 302 (75%) participated in the study, 27 (7%) refused interview, 66 (16%) were unable to be contacted, and 7 (2%) were excluded because of language difficulties. In univariate analysis, there was no significant difference between participating patients and nonparticipating patients with respect to sex (p = 0.226), geographic location of residence (p = 0.341), indigenous status (p = 0.123), length of stay in hospital (p = 0.477), or ventilation status (p = 0.890). However, interviewed patients were significantly younger (median 45 years, range 16–88 years) than patients who were not interviewed (median 51 years, range 17–88 years) (p = 0.007).

Of 1,252 potential controls, 603 (48%) participated in the study. Of those remaining, 357 (28%) refused to participate, 153 (12%) were excluded because of language difficulties, and 139 (11%) were unavailable for interview. Of the controls, 25% were ages 16–35 years, 36% were 36–55 years, and 37% were >55 years. This compares with the adult metropolitan Sydney population of 38% ages 16–35 years, 36% 36–55 years, and 28% >55 years (Australian Bureau of Statistics, unpub. data).

### Descriptive Epidemiology

The median age of case-patients was 45 years (range 16–88 years). Of the 302 case-patients, 125 (41%) were male, 68 (23%) were admitted to a high dependency or intensive care unit, and 37 (12%) required mechanical ventilation. The median length of hospital stay was 4 nights (range 1–91 nights). Reported risk factors among case-patients included a history of asthma (38%, including 29% who required regular medication), lung disease (19%), diabetes (19%), mental health diagnosis (18%), heart disease (14%), pregnancy (13%), obstructive sleep apnea (12%), immune suppression (10%), neurologic condition (8%), liver disease (8%), kidney disease (3%), blood disorders (5%), and metabolic conditions (1%) (Table A1).

None of the case-patients reported giving birth in 28 days before symptom onset. Of the 40 patients who were pregnant at the time of symptom onset, 28 (70%) were in their third trimester. Sixty-six (22%) case-patients were current smokers, and 91 (30%) were ex-smokers. Among the 91 ex-smokers, the median time smoked was 20 years (range 1–60 years, median 15 cigarettes/day); 39 reported cessation >5 years prior to illness, 15 patients between 12 months and 5 years, and 37 patients within the past 12 months. Among the 66 current smokers, the median time smoked was 20 years (range 1–60 years; median 10 cigarettes/day) (online Appendix Table).

### Case–Control Study

There were no significant differences in characteristics of case-patients and controls by place of residence, receipt of 2009 seasonal influenza vaccination, or history of neurologic disorders (Table A1). In univariate analysis, compared with controls, case-patients were more likely to be male, aged 16–35 years and 46–55 years, have higher body mass index (BMI), and report a history of asthma, heart disease, mental health diagnosis, immune suppression, obstructive sleep apnea, lung disease, diabetes, liver disease, blood disorder, and pregnancy, and smoking (Table A1).

In the logistic regression model, age, sex, asthma (requiring regular medication), smoking (current or ex-smoker), heart disease, immune suppression, lung disease, diabetes, and pregnancy were independently associated with hospitalization for pandemic (H1N1) 2009 (Table 1). Similar results were found when analysis was restricted to men only or women only. The factor most strongly associated with hospitalization was pregnancy, followed by lung disease, immune suppression, and ages 16–25 and 46–55 years (Table 1). Overall, 262 (86%) case-patients reported >1 independent risk factor (asthma, heart disease, immune suppression, lung disease, diabetes, pregnancy, or smoking) compared with 315 (52%) of controls (p<0.0001). The risk for hospitalization increased with increasing number of reported significant risk factor*s*.

**Table 1:** Independent risk factors for hospitalization from pandemic (H1N1) 2009 influenza, all case-patients and controls, Sydney, Australia, 2009*

Patient characteristic	Adjusted OR (95% CI)	p value
Sex		
F	Referent	
M	1.8 (1.2–2.5)	0.0017
Age, y		
16–25	5.4 (2.5–11.4)	
26–35	4.1 (2.0–8.3)	
36–45	3.9 (2.0–7.6)	<0.0001
46–55	5.1 (2.7–9.6)	
56–65	1.9 (1.0–2.5)	
>65	Referent	
Underlying condition		
Asthma, regular medication	4.3 (2.7–6.8)	<0.0001
Heart disease	2.3 (1.2–4.1)	0.0083
Immunosuppression	5.5 (2.8–10.9)	<0.0001
Lung disease	6.6 (3.8–11.6)	<0.0001
Diabetes	3.8 (2.2–6.5)	<0.0001
Pregnancy	22.4 (9.2–54.5)	<0.0001
Smoking status		
Nonsmoker	Referent	
Current smoker	2.0 (1.3–3.2)	0.002
Former smoker	2.0 (1.3–3.0)	
No. significant risk factors		
0	Referent	
1	3.9 (2.6–5.8)	
2	9.3 (5.9–14.6)	<0.0001
3	20.4 (10.4–40.2)	
>4	80.3 (10.1–638.7)	

*OR, odds ratio; CI, confidence interval.

In the logistic regression model for women of childbearing age, asthma (requiring regular medication), lung disease, diabetes, and pregnancy were independently associated with hospitalization for pandemic (H1N1) 2009 (Table 2). In total, 88% of women of childbearing age hospitalized for pandemic (H1N1) 2009 reported 1 of the risk factors compared with 39% of controls (p <0.0001). Pregnancy was the only risk factor reported for 25% of hospitalized women of childbearing age.

**Table 2:** Risk factors for hospitalization caused by pandemic (H1N1) 2009 influenza for female case-patients and controls of childbearing age, Sydney, Australia, 2009*

Patient characteristic	No. (%) case-patients, n = 99	No. (%) controls, n = 181	OR (95% CI)	p value	Adjusted OR (95% CI)	p value
Age, y						
16–25	28 (28)	43 (24)	1.4 (0.8–2.6)			
26–35	37 (37)	64 (35)	1.3 (0.7–2.2)	0.5233		
36–45	34 (34)	74 (41)	Referent			
Aboriginal status						
Nonindigenous	95 (98)	177 (96)	Referent	0.3864		
Indigenous	4 (4)	4 (2)	1.9 (0.5–7.6)			
Body mass index†						
Underweight (<18.5)	8 (9)	9 (5)	2.8 (1.0–8.0)			
Normal (18.5–24.9)	31 (33)	99 (57)	Referent			
Overweight (25.0–29.9)	15 (16)	35 (20)	1.4 (0.7–2.8)	0.0003		
Obese 1 (30.0–34.9)	17 (18)	18 (10)	3.0 (1.4–6.6)			
Obese 2 (35.0–39.9)	10 (11)	4 (2)	8.0 (2.3–27.3)			
Obese 3 (>40)	12 (13)	8 (5)	4.8 (1.8–12.8)			
Health condition‡						
Asthma	46 (46)	35 (19)	Referent			
No regular medication	7 (7)	21 (12)	0.9 (0.4–2.3)	<0.001	10 (4.0–21.0)	<0.0001
Regular medication	39 (39)	14 (8)	7.7 (3.9–15.3)			
Heart disease§	0	1 (0.5)	<0.001	0.9871		
Kidney disease	0	1 (0.5)	<0.001	0.987		
Mental health problem	17 (17)	19 (11)	1.8 (0.9–3.6)	0.1139		
Neurologic problem	4 (4)	8 (4)	0.9 (0.3–3.1)	0.8809		
Immunosupression	1 (1)	2 (1)	0.9 (0.1–10.2)	0.942		
Obstructive sleep apnea	10 (10)	4 (2)	5.0 (1.5–16.3)	0.0081		
Lung disease	10 (10)	4 (2)	5.0 (1.5–16.3)	0.0081	7 (2.0–28.0)	0.0043
Diabetes	14 (14)	2 (1)	14.7 (3.3–66.3)	0.0005	20 (4.0–103.0)	0.0003
Metabolic disorder	1 (1)	2 (1)	0.9 (0.1–10.2)	0.942		
Liver disease	5 (5)	0	>999.9	0.9798		
Blood disorder	4 (4)	8 (4)	0.9 (0.3–3.1)	0.8809		
Pregnancy	40 (40)	7 (4)	16.9 (7.2–39.6)	<0.001	28 (11.0–70.0)	<0.0001
Smoking status						
Nonsmoker	53 (54)	130 (72)	Referent			
Current smoker	24 (24)	32 (18)	1.8 (1.0–3.4)	0.0057		
Former smoker	22 (22)	19 (11)	2.8 (1.4–5.7)			
Influenza vaccine in 2009¶	20 (20)	30 (17)	1.3 (0.7–2.4)	0.4262		

*OR, odds ratio; CI, confidence interval. †8 controls and 6 case-patients missing data. ‡Groups are not mutually exclusive. §1 control and 3 case-patients missing data. ¶1 case-patient missing data.

In univariate analysis, compared with controls, case-patients who required mechanical ventilation were more likely to report a history of lung disease, asthma (requiring regular medication), have a higher BMI, be pregnant, have an influenza vaccination in the previous 12 months, and be 26–45 years of age (Table 3). In the logistic regression model for ventilated case-patients, a history of lung disease, diabetes, pregnancy, high BMI, or status as a current or ex-smoker were independently associated with mechanical ventilation (Table 3).

**Table 3:** Risk factors for mechanical ventilation because of pandemic (H1N1) 2009 infection, Sydney, Australia, 2009*

Patient characteristic	No. (%) case-patients, n = 37	No. (%) controls, n = 603	OR (95% CI)	p value	Adjusted OR (95% CI)	p value
						
Sex						
M	11 (30)	207 (34)				
F	26 (70)	396 (66)	1.2 (0.6–2.6)	0.5674		
Age, y						
16–25	3 (8)	61 (10)	3.7 (0.7–19)		5.5 (0.7–42.0)	
26–35	11 (30)	92 (15)	9.1 (2.5–33.3)		12.2 (2.5–58.2)	
36–45	12 (32)	111 (18)	8.2 (2.3–29.7)		10.4 (2.3–48.3)	
46–55	8 (22)	111 (18)	5.5 (1.4–21.0)		10.2 (2.3–45.9)	
>55	3 (8)	228 (38)		0.0114		0.0176
Aboriginal status						
Nonindigenous	36 (97)	596 (99)				
Indigenous	1 (3)	7 (1)	2.4 (0.3–19.7)	0.426		
Body mass index†						
Underweight (<18.5)	3 (9)	15 (3)	8.6 (2.0–37.9)		10.6 (1.9–58.9)	
Normal (18.5–24.9)	6 (17)	259 (45)		0.0004		0.0022
Overweight (25.0–29.9)	10 (29)	175 (30)	2.5 (0.9–6.9)		3.2 (1.0–10.3)	
Obese 1 (30.0–34.9)	5 (14)	79 (14)	2.7 (0.8–9.2)		1.8 (0.5–7.4)	
Obese 2 (35.0–39.9)	5 (14)	29 (5)	7.4 (2.1–25.9)		9.8 (2.4–40.1)	
Obese 3 (>40)	6 (17)	21 (4)	12.3 (3.7–41.6)		11.6 (2.7–49.3)	
Health condition‡						
Asthma	13 (65)	93 (15)				
No regular medication	5 (14)	48 (8)	2.2 (0.8–6.1)	0.0061		
Regular medication	8 (22)	45 (7)	3.8 (1.6–8.9)			
Heart disease§	3 (9)	41 (7)	1.3 (0.4–4.3)	0.7136		
Kidney disease¶	1 (3)	8 (1)	2.1 (0.3–16.9)	0.5013		
Mental health problem	6 (16)	49 (8)	2.2 (0.9–5.5)	0.0958		
Neurologic problem	3 (8)	42 (7)	1.2 (0.3–4.0)	0.792		
Immunosuppression	1 (3)	22 (4)	0.7 (0.1–5.6)	0.7655		
Obstructive sleep apnea	2 (5)	23 (4)	1.4 (0.3–6.4)	0.6296		
Lung disease	6 (16)	31 (5)	3.6 (1.4–9.2)	0.0084	8.6 (2.6–28.5)	0.0005
Diabetes	5 (14)	38 (6)	2.3 (0.9–6.3)	0.0977	4.4 (1.2–15.6)	0.0383
Liver disease	2 (5)	20 (3)	1.7 (0.4–7.4)	0.503		
Pregnancy	8 (22)	7 (1)	23.4 (8.0–69.2)	<0.001	40.5 (9.7–168.1)	<0.0001
Smoking status						
Nonsmoker	17 (46)	372 (62)			2.4 (1.0–5.5)	0.0325
Current or former smoker	20 (54)	231 (38)	1.9 (1.0–3.7)	0.0605		
Influenza vaccine in 2009	20 (54)	202 (34)	0.3 (0.1–0.8)	0.0163		

*OR, odds ratio; CI, confidence interval. †Data missing for 2 case-patients and 25 controls. ‡Groups are not mutually exclusive. §Date missing for 2 case-patients and 12 controls.  ¶Data missing for 3 controls.

Of the 603 controls, 113 (19%) reported an influenza-like-illness during the study period. There was no significant difference in underlying risk factors between controls who reported influenza-like-illness and controls who did not report any respiratory symptoms. However, influenza-like-illness was significantly more common among controls aged 16–25 (odds ratio [OR] 3.3, 95% confidence interval [CI] 1.4–7.5, p = 0.005) and 36–45 years (OR 3.0, CI 1.4–6.2, p = 0.004) compared with controls aged >65 years.

Information on the time from onset of illness to medical attention was available for 295 (98%) case-patients. Of these, 238 (81%) reported having >1 risk factor listed in the public message campaigns. Overall, the proportion of case-patients seeking medical attention was similar for both those with reported risk factors and those with no risk factors (80% and 79%, respectively). There was no significant difference in individual underlying risk factors between case-patients who sought medical attention within 48 hours of symptoms and those who did not (Table 4).

**Table 4:** Comparison of characteristics of patients with cases of pandemic (H1N1) 2009 influenza by time from symptom onset to medical attention, Sydney, Australia, 2009*

Patient characteristic	Medical attention within 48 h		OR (95% CI)	p value
Yes, no. (%) patients, n = 235	No, no. (%) patients, n = 60
Sex				
M	97 (41)	24 (40)		
F	138 (59)	36 (60)	1.1 (0.6–1.9)	0.8576
Age, y				
16–25	33 (14)	5 (8)	1.1 (0.3–4.3)	
26–35	44 (19)	9 (15)	0.8 (0.3–2.8)	
36–45	47 (20)	11 (18)	0.7 (0.2–2.3)	
46–55	50 (21)	20 (33)	0.4 (0.1–1.3)	
56–65	32 (14)	10 (17)	0.6 (0.2–1.8)	
>65	29 (12)	5 (8)		0.3677
Aboriginal status				
Nonindigenous	229 (97)	58 (97)		0.7409
Indigenous	6 (3)	2 (3)	0.8 (0.1–3.9)	
Body mass index†				
Underweight (<18.5)	13 (6)	2 (3)	1.6 (0.3–7.8)	
Normal (18.5–24.9)	65 (28)	16 (27)		0.4457
Overweight (25.0–29.9)	67 (29)	10 (17)	1.6 (0.7–3.9)	
Obese 1 (30–34.9)	38 (16)	12 (20)	0.8 (0.3–1.8)	
Obese 2 (35–39.9)	23 (10)	6 (10)	0.9 (0.3–2.7)	
Obese 3 (>40)	19 (8)	8 (13)	0.6 (0.2–1.6)	
Health condition‡				
Asthma	89 (38)	24 (40)		0.3607
No regular medication	16 (7)	9 (15)		
Regular medication	73 (31)	15 (25)	1.4 (0.7–2.6)	
Heart disease§	31 (13)	7 (12)	1.2 (0.5–2.8)	0.703
Kidney disease	8 (3)	2 (3)	1.0 (0.2–4.9)	0.9785
Mental health problem	34 (14)	17 (28)	0.4 (0.2–0.8)	0.0129
Neurologic problem	14 (6)	8 (13)	0.4 (0.2–1.0)	0.0586
Immunosuppression	22 (9)	6 (10)	0.9 (0.4–2.4)	0.8803
Obstructive sleep apnea	26 (11)	10 (17)	0.6 (0.3–1.4)	0.2399
Lung disease	46 (20)	11 (18)	1.1 (0.5–2.2)	0.828
Diabetes	48 (20)	10 (17)	1.3 (0.6–2.7)	0.5141
Liver disease	16 (7)	5 (8)	0.8 (0.3–2.3)	0.6823
Pregnancy	33 (14)	7 (12)	1.3 (0.5–3.2)	0.6008
Smoking status¶				
Nonsmoker	113 (48)	26 (43)		
Current or former smoker	121 (51)	34 (57)	0.8 (0.5–1.5)	0.493
Influenza vaccine in 2009#	86 (37)	19 (32)	1.3 (0.7–2.3)	0.4507

*OR, odds ratio; CI, confidence interval. †Data missing for 10 case-patients who received medical attention within 48 h and 6 who did not. ‡Groups are not mutually exclusive. §Data were missing for 6 case-patients who received medical attention within 48 h. ¶Data were missing for 1 case-patient who received medical attention within 48 h. #Vaccination information was missing for 2 case-patients and 1 control.

## Discussion

We found that pregnancy, lung disease, immune suppression, asthma, diabetes, heart disease, and a history of smoking were associated with hospitalization from pandemic (H1N1) 2009 infection. Among women of childbearing age, pregnancy was the single greatest risk factor for hospitalization, followed by diabetes, history of asthma requiring regular medication, and lung disease. Obesity was not an independent risk factor for hospitalization although it was a risk factor for mechanical ventilation. The majority of case-patients sought medical attention within 48 hours. This study did not identify any particular risk groups that were less likely to seek early medical attention.

Our study was designed to identify risk factors for moderate to severe illness resulting from influenza (as measured by requirement for hospital admission), not risk factors for acquiring influenza. Controls for the study were therefore selected from the community rather than nonhospitalized case-patients. When controls who reported influenza-like-illness were compared with controls without these symptoms, there was no significant difference in risk factors other than age. This finding suggests that apart from age (which is likely to reflect past infection with influenza strains that protected against pandemic [H1N1] 2009) (*25*), other participant characteristics were not important for determining susceptibility for infection.

Our data are subject to several limitations. First, compared with the adult population in Sydney, controls were older and a higher proportion were women, introducing the possibility of bias if the groups were not otherwise similar. However, our analysis adjusted for age and sex, and the findings were consistent with those of similar studies examining risk factors for seasonal influenza (*3*–*5*,*6*). When the model was restricted to gender, the results were similar to the final model. These findings suggest that the potential bias from control selection on the final model was minimal. Second, risk factor status was determined by self-report for case-patients and controls. However, public messaging during the pandemic relied on the persons recognizing that they were in a high-risk group. For this reason, self-reported risk factors were thought to be a good indication of identified risk. However, undiagnosed or unacknowledged medical conditions would not have been captured in our study and could underestimate the effect of some risk factors. Third, the underlying reason for admission to hospital for patients (whether specifically caused by the infection or because of preexisting illness or both) was not determined in this study. Case-patients were selected for inclusion in the study if they met the pandemic (H1N1) 2009 case definition, were hospitalized, and had onset of symptoms >2 days before admission. Fourth, although pregnancy was identified as an independent risk factor for hospitalization, the magnitude of this risk may be biased upward if clinicians had a lower threshold for admitting pregnant women as a precaution, particularly in later stages of pregnancy. Fifth, patients who died following pandemic (H1N1) 2009 infection were excluded from the study, thereby excluding those with the most severe disease. Although patients who died were obviously unable to be interviewed, information regarding the presence of underlying medical conditions was collected from the treating clinicians. During the study period, there were 23 deaths that met the study’s case definition. Similar to interviewed case-patients, 20 (87%) of the patients who died were reported to have >1 significant risk factor. However, a higher proportion of patients who died were reported as having lung disease (60% vs. 19%), being a current smoker (35% vs. 22%), and having immune suppression (25% vs. 10%) when compared with study participants. Deceased patients were significantly older (median 59 years, range 23–85 years) compared with surviving patients (median 45 years, range 16–88 years) included in the study (p<0.001).

Descriptive studies of pandemic (H1N1) 2009 infection have reported obesity, heart disease, diabetes, pregnancy (*26*–*28*), kidney disease, neurologic disease, immune suppression, lung disease, asthma, smoking, and relatively young age (*29**,**30*) as the most common concurrent conditions for hospitalized patients (*11*–*20*). Given that many of these underlying medical conditions do not occur in isolation, our analytical study was able to ascertain which of these were independently associated with hospitalization from pandemic (H1N1) 2009.

Although high rates of pandemic (H1N1) 2009 infection have been reported from indigenous people in other reports (*31*), our study lacked sufficient power to explore the independent impact of being Aboriginal on the risk factor of moderate to severe disease. However, among those Aboriginal case-patients included, all reported a history of other independent risk factors. These data may suggest that it is the high prevalence of risk factors for severe disease that place Aboriginal people at an increased risk rather than genetic susceptibility.

BMI was not independently associated with increased risk for hospitalization with pandemic (H1N1) 2009. Obesity appears to be a confounder of other risk factors for overweight patients. Of the 58 case-patients with BMI >35 (very obese), 55 (95%) reported >1 significant risk factor, including smoking (35/55, 64%), asthma (32/55, 58%), and diabetes (18/55, 33%). Further analysis suggested that obesity was independently associated with increased risk of ventilation in our study. Of the 35 ventilated case-patients, 11 reported a BMI >35, and all but 2 patients reported other significant risk factors.

Our study highlights the increased risk of moderate to severe illness from pandemic (H1N1) 2009 for pregnant women and introduces smoking as an independent risk factor for hospitalization from pandemic (H1N1) 2009. In addition, our study provides evidence to support the continuation of influenza prevention efforts (including vaccination) targeted to persons with lung disease, immune suppression, asthma, diabetes, and heart disease. Although Aboriginal status and obesity may not be independent risk factors for severe disease, they indicate the likely presence of other risk factors, and so prevention messages should continue to be directed to these groups.

## Appendix

**Table A1:** Comparison of characteristics for case-patients and controls in a study of risk for hospitalization because of pandemic (H1N1) 2009 influenza, Sydney, Australia, 2009*

Variable	No. (%) case-patients, n = 302	No. (%) controls, n = 603		
			OR (95% CI)	p value
Sex				
F	177 (59)	396 (66)	Referent	
M	125 (41)	207 (34)	1.4 (1.0–1.8)	0.0379
Age, y				
16–25	40 (13)	61 (10)	2.4 (1.4–4.1)	
26–35	54 (18)	92 (15)	2.1 (1.3–3.5)	
36–45	58 (19)	111 (18)	1.9 (1.2–3.1)	
46–55	72 (24)	111 (18)	2.3 (1.4–3.8)	
56–65	44 (15)	105 (17)	1.5 (0.9–2.5)	
>65	34 (11)	123 (20)	Referent	0.007
Area of residence in Sydney				
South East	34 (11)	71 (12)		
West	112 (37)	229 (38)		
South West	123 (41)	239 (40)		
North	33 (11)	64 (11)		
Aboriginal status				
Nonindigenous	293 (97)	596 (99)	Referent	
Indigenous	9 (3)	7 (1)	2.6 (1–7.1)	0.0591
Body mass index†				
Underweight (<18.5)	15 (5)	15 (3)	3.0 (1.4–6.5)	<0.0001
Normal (18.5–24.9)	85 (30)	259 (45)	Referent	
Overweight (25.0–29.9)	77 (27)	175 (30)	1.3 (0.9–1.9)	
Obese 1 (30–34.9)	51 (18)	79 (14)	2.0 (1.3–3.0)	
Obese 2 (35–39.9)	31 (11)	29 (5)	3.3 (1.9–5.7)	
Obese 3 (>40)	27 (10)	21 (4)	3.9 (2.1–7.3)	
Concurrent conditions‡				
Asthma	114 (38)	93 (15)		
No regular medication	25 (8)	48 (8)	1.4 (0.8–2.4)	<0.0001
Regular medication	89 (29)	45 (7)	5.4 (3.6–8.0)	
Previous hospitalization	48 (16)	24 (4)		
Heart disease§	40 (14)	41 (7)	2.1 (1.3–3.3)	0.0016
Heart failure	8 (20)	1 (2)		
Angina	10 (25)	7 (17)		
Heart attack	8 (20)	9 (22)		
Congenital condition	6 (15)	4 (10)		
Rheumatic fever	6 (15)	1 (2)		
Kidney disease¶	10 (3)	8 (1)	2.5 (1.0–6.5)	0.0522
Renal failure	5 (50)	4 (50)		
Dialysis	3 (30)	–		
Transplant or waiting list	4 (40)	–		
Mental health problem	53 (18)	49 (8)	2.4 (1.6–3.6)	<0.0001
Neurologic problem	24 (8)	42 (7)	1.2 (0.7–1.9)	0.5925
Immunosuppression	30 (10)	22 (4)	2.9 (1.6–5.1)	0.0002
Obstructive sleep apnea	37 (12)	23 (4)	3.5 (2.1–6.0)	<0.0001
Lung disease	58 (19)	31 (5)	4.4 (2.8–7.0)	<0.0001
Regular medication	39 (67)	13 (42)		
Previous hospitalization	32 (55)	11 (35)		
Diabetes	58 (19)	38 (6)	3.5 (2.3–5.5)	<0.0001
Metabolic disorder	3 (1)	7 (1)	0.9 (0.2–3.3)	0.8204
Liver disease	23 (8)	20 (3)	2.4 (1.3–4.4)	0.0053
Blood disorder	16 (5)	16 (3)	2.1 (1.0–4.2)	0.0463
Pregnancy	40 (13)	7 (1)	13.0 (5.7–29.4)	<0.0001
First trimester	5 (13)	2 (18)		
Second trimester	7 (18)	3 (27)		
Third trimester	28 (70)	2 (18)		
Smoking status#				
Nonsmoker	144 (48)	372 (62)	Referent	
Current smoker	66 (22)	82 (14)	2.1 (1.4–3.0)	0.0002
Former smoker	91 (30)	149 (25)	1.6 (1.1–2.2)	
Influenza vaccine in 2009**	107 (36)	202 (34)	1.1 (0.8–1.5)	0.529

*OR, odds ratio; CI, confidence interval. †Groups are not mutually exclusive.  ‡Body mass index calculated as weight in kilograms divided by height in meters squared. Data were missing for 16 case-patients and 25 controls. §Data were missing for 6 case-patients and 12 controls. ¶Data were missing for 2 controls.  #Data were missing for 2 case-patients and 2 controls. **Vaccination information was missing for 2 case-patients and 1 control.
